# Relationship between radiation dose and microbleed formation in patients with malignant glioma

**DOI:** 10.1186/s13014-017-0861-5

**Published:** 2017-08-10

**Authors:** Michael Wahl, Mekhail Anwar, Christopher P. Hess, Susan M Chang, Janine M. Lupo

**Affiliations:** 1 Department of Radiation Oncology, University of California, San Francisco, CA USA; 2 Department of Radiology and Biomedical Imaging, University of California, San Francisco, CA USA; 3 Department of Neurology, University of California, San Francisco, CA USA; 4 Department of Neurological Surgery, University of California, San Francisco, CA USA

**Keywords:** Glioma, Microbleeds, Radiation therapy, Susceptibility-weighted imaging, Treatment effects

## Abstract

**Background:**

Cranial irradiation is associated with long-term cognitive changes. Cerebral microbleeds (CMBs) have been identified on susceptibility-weighted MRI (SWI) in patients who have received prior cranial radiation, and serve as radiographic markers for microvascular injury thought to contribute to late cognitive decline. The relationship between CMB formation and radiation dose has not previously been quantified.

**Methods:**

SWI was performed on 13 patients with stable WHO grade III-IV gliomas between 2 and 4 years after chemoradiotherapy to 60 Gy. The median age at the time of treatment was 41 years (range 25 – 74 years). CMBs were identified as discrete foci of susceptibility on SWI that did not correspond to vessels. CMB density for low (<30 Gy), median (30–45 Gy), and high (>45 Gy) dose regions was computed.

**Results:**

Twelve of 13 patients exhibited CMBs. The number of CMBs was significantly higher for late (>3 years from treatment) compared to early (<3 years) timepoints (early median 6 CMBs; late median 27 CMBs; *p* = 0.001), and there were proportionally more CMBs at lower doses for late scans (*p* = 0.006). 88% of all CMBs were observed in regions receiving at least 30 Gy, but the CMB density within medium and high dose regions was not significantly different (*p* = 0.33 and *p* = 0.9, respectively, for early and late time points).

**Conclusions:**

CMBs predominantly form in regions receiving at least 30 Gy, but form in lower dose regions with longer follow-up. We do not observe a clear dose–response relationship at doses above 30 Gy. These findings provide important information to assess the risk of late microvascular sequelae from cranial irradiation.

## Introduction

Radiotherapy plays an integral role in the management of gliomas. Combined adjuvant chemoradiotherapy (CRT) constitutes the standard of care after maximal safe resection in malignant glioma [1], and is gaining acceptance as adjuvant treatment for high-risk, low-grade gliomas [2]. Due to pathologic studies demonstrating microscopic spread beyond tumor visible on imaging [3–5], radiotherapy targets generally constitute a margin of approximately 2 cm around gross visible disease and surgical resection cavities. Thus, even with modern conformal radiotherapy techniques, a significant amount of normal brain tissue receives the prescription dose of 60 Gy, with an even larger surrounding region irradiated to lower doses [6].

Given the amount of normal irradiated brain tissue, late toxicity from radiotherapy for gliomas is of significant concern, particularly for patients with lower-grade gliomas for whom survival can extend well over 10 years [2]. Cognitive testing of long-term glioma survivors has shown significant decline across multiple cognitive domains [7], although evidence exists for tumor progression as a driver of cognitive decline in glioma patients [8]. Reliable markers of late radiation toxicity are needed to better characterize the mechanism of white matter damage as well as patient, tumor, and treatment factors that influence the risk of late cognitive decline.

Studies with primate [9] and murine models [10], as well as human autopsy studies [11, 12], have demonstrated that microvascular damage is a prominent pathologic component of radiation-induced brain injury, with endothelial degeneration leading to the formation cerebral microbleeds (CMBs). CMBs have also been observed in elderly patients without known underlying pathology [13, 14], as well as in other degenerative central nervous system conditions, including cerebral amyloid angiopathy (CAA) associated with Alzheimer’s disease and vascular cognitive impairment [11], where they have been linked to cognitive dysfunction [15–18]. Thus, quantification of CMB characteristics may serve as a valuable metric for radiation-induced vasculopathy and resultant cognitive decline.

Susceptibility-Weighted Imaging (SWI) is a powerful, noninvasive tool for detecting hemosiderin-containing CMBs, and has been utilized in several studies of stroke and vascular injury [19–24]. Previous studies with SWI in patients with gliomas demonstrated that CMBs appeared in irradiated patients starting approximately two years after treatment, with an increasing number of lesions over time; in contrast, patients with gliomas who did not receive radiotherapy did not develop CMBs [25]. Early CMBs were generally observed in the area of relatively high-dose radiation, while CMBs that appeared later extended to lower-dose regions, including contralateral cerebral hemispheres. Administration of anti-angiogenic therapy during the course of radiation has also been shown to decrease the rate of CMB formation [26]. Finally, the formation of CMBs has been associated with neurocognitive dysfunction in both pediatric and adult patients undergoing radiotherapy [27, 28].

While these studies have suggested an association between radiation dose and CMB formation [29–31], the precise quantitative relationship between radiation dose and the risk of CMB formation is largely uncharacterized. The purpose of this study was to directly relate CMB characteristics to radiation dose received.

## Materials and methods

### Patients and treatment

Thirteen patients with newly diagnosed malignant gliomas were included in this retrospective study. 10 patients had histologically confirmed WHO grade IV glioblastoma, while the remaining 3 patients had WHO grade III gliomas (1 anaplastic astrocytoma, 1 anaplastic oligodendroglioma, 1 anaplastic oligoastrocytoma). Median patient age at the time of radiotherapy was 41 years (range 25 – 74 years). All patients underwent resection followed by focal radiotherapy to a total dose of 60 Gy in 30 daily fractions to the gross residual tumor and surgical resection cavity, with a margin determined by the treating physician but typically approximately 2 cm, and received concurrent and adjuvant temozolomide; six patients underwent treatment with additional investigational agents, including enzastaurin (4 patients) or bevacizumab and erlotinib (2 patients). Single time point or serial MR imaging with SWI was performed approximately 2 to 4 years after treatment (median 27 months; range 22 – 52 months), for a total of 18 MRIs, with 3 of the 13 patients undergoing multiple scans. One patient was excluded from analysis due to the early formation of numerous microbleeds away from the radiotherapy region felt to be most consistent with an alternate etiology. All patients provided informed consent in accordance with guidelines established by our institutional review board.

### MRI acquisition

SWI was performed on a GE whole-body 3 T scanner (GE Healthcare Technologies, Milwaukee, WI) with volume excitation and eight-channel phased-array reception (Nova Medical, Wilmington, MA). A three-dimensional (3D) spoiled gradient echo (SPGR) sequence was applied with echo time/repetition time (TE/TR) of 28 ms/56 ms, flip angle 20°, 24 × 24 cm^2^ field of view, and 2 mm slice thickness. To keep the scan time under 7 min, a generalized autocalibrating partially parallel acquisition was used with a two-fold reduction factor, 512 × 144 acquired matrix, 0.5 × 0.5-mm reconstructed in-plane resolution, and 16 autocalibrating lines [32]. Standard clinical pre- and post-gadolinium T1-weighted 3D SPGR and T2-weighted fluid attenuated inversion recovery images were acquired for anatomic comparison.

### Data processing and analysis

The complex k-space data from all eight channels of the 3 T SWI scan were transferred off-line, and postprocessing was performed using in-house programs developed with MATLAB 12.0 software (MathWorks, Natick, MA) on a Linux cluster. Standard SWI postprocessing was performed on the reconstructed k-space data for each coil, which were then combined, intensity-corrected, and projected through 8-mm-thick slabs [21]. Phase images were created as previously described by our group [33].

For all patients, radiation dosimetry maps were retrieved on a Pinnacle treatment planning system (Phillips Medical Systems, Andover MA). The non-contrast CT scan obtained for radiotherapy treatment planning was aligned to the SWI image using affine registration after skull stripping of both images [34, 35]. This transformation was then applied to the radiation dosimetry map to allow registration of radiation dose to the SWI.

CMBs were manually identified on SWI as discrete foci of susceptibility that did not correspond to vessels or surgical cavity on consecutive axial slices. There were no type I-III cavernomas seen in our analysis. Although it is possible that type IV cavernomas were included in the analysis, as the mechanism behind type IV cavernoma formation is thought to be similar to microbleeds, this is unlikely since all microbleeds observed in this study were less than 5 mm in diameter and from our prior experience most do not grow larger than that as far as 15 years post –RT. Filtered phase images were used to confirm the absence of calcifications from the analysis. The number of microbleeds was counted in normal-appearing tissue, outside the contrast-enhancing tumor region and any areas of acute hemorrhage. In order to minimize user error from counting, microbleeds from each dataset were labeled and iteratively counted multiple times, until the same number of counts was obtained from 2 consecutive trials as performed by Lupo et al. [25]. The maximum number of iterations required for all patients was 4, and the counter was blinded to the date of RT.

For each scan, the number of labeled CMBs was counted in multiple regions: within the entire SWI volume, within prespecified radiation dose regions (low: <30 Gy, medium: 30–45 Gy and high: >45 Gy), and within each cortical lobe as defined using the standardized Montreal Neurologic Institute (MNI) atlas [36]. Microbleed Density (MD) was then calculated as the ratio of the total number of CMBs within a doismetric region to the total volume of that region, excluding areas of surgical cavity or contrast enhancement. To account for dose heterogeneity between cortical lobar regions, the ratio of total number of CMBs within a lobar region to the volume of that region receiving at least 30 Gy was calculated (MD_V30_). Dosimetric parameters including the brain volume receiving at least 30, 45 and 60 Gy (V30, V45 and V60, respectively) were calculated and correlated with CMB count. For analysis, scans were grouped according to timing of scan after treatment, with scans under 3 years characterized as “early” (9 patients, 9 scans, median time after treatment 2.0 years), while all scans ≥3 years as “late” (5 patients, 7 scans, median time after treatment 3.5 years). Two patients underwent scans at both the 3 and 4 year timepoints; both scans were included in the analysis.

### Statistical design

Comparisons of CMB counts and densities between timepoints and categorical patient variables were performed using a Wilcoxon rank-sum test, while correlations between CMB counts and continuous patient variables were performed using a Spearman’s ρ. The Kolmogorov-Smirnov test was used to evaluate for a difference between the distribution of CMBs as a function of dose as a continuous variable at early and late timepoints, while the Chi squared test was used to evaluate the relative frequency of CMBs in low, medium and high-dose regions at early and late timepoints. Differences in distribution of CMBs within cortical lobes between early and late timepoints were evaluated using a Kruskal-Wallis test.

## Results

### Lesion counts

**Fig. 1:**
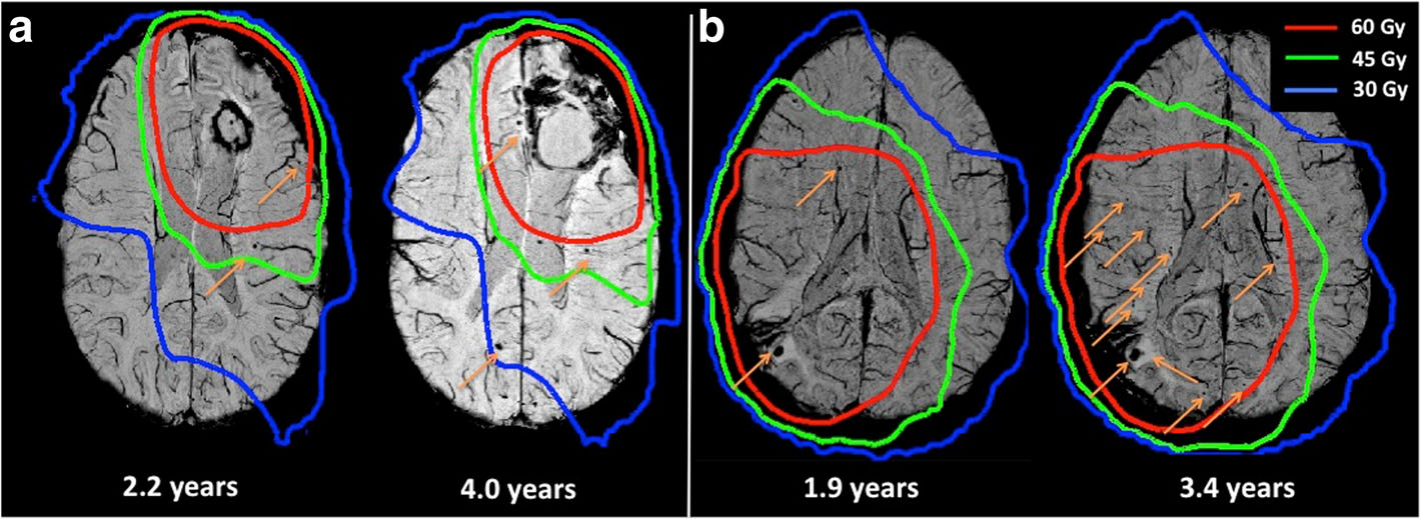
Example Microbleeds. (**a**) and (**b**) demonstrate example patients with SWI images obtained at early (*left*) and late (*right*) timepoints, demonstrating progression in the number and distribution of CMBs between scans

Example CMBs are shown in Fig. 1. CMBs were observed in all but one patient, with a median CMB count of 9 (range: 0 and 70). A total of 306 CMBs were observed (Table 1). The number of CMBs was observed to increase with time, with patients scanned after 3 years demonstrating significantly more CMBs than those scanned at or before 3 years (median CMB count: 27 vs 6; *p* = 0.001). The three patients who underwent scans at both timepoints all demonstrated substantially increased CMB counts with the later scan (25, 25 and 71 vs 4, 2 and 21). For both early and late timepoints, CMB count was not associated with patient age (early: Spearman’s ρ = −0.109, *p* = 0.78; late: Spearman’s ρ = 0.514, *p* = 0.24) or the use of anti-angiogenic therapy (early: *p* = 0.81; late: *p* = 0.48).

**Table 1:** Summary of total CMB counts and densities across all subjects by anatomic and dosimetric region. For lobar regions, density calculations are normalized to the volume of each lobe receiving at least 30 Gy (MD_V30_). Units of CMB density are given in CMBs/dm^3^

	1-2 Years		3-4 Years	
Region	Total CMB Count	Median CMB Density (range)	Total CMB Count	Median CMB Density (range)
Whole Brain	66	4.43 (0.68 – 12.4)	240	23.0 (15.5 - 47.4)
<30 Gy	2	0 (0–1.48)	35	6.41 (2.48 – 14.6)
30-45 Gy	8	5.7 (0 – 14.1)	52	32.9 (28.9 – 74.7)
>45 Gy	56	9.51 (0 – 27.0)	154	47.6 (24.8 – 97.6)
Frontal Lobe	6	0 (0–6.30)	50	29.1 (0.0 – 70.3)
Parietal Lobe	19	8.27 (0–45.9)	58	75.3 (26.9 – 135)
Occipital Lobe	6	0 (0 – 38.1)	35	37.9 (0 – 97.8)
Temporal Lobe	9	5.10 (0 – 30.7)	26	67.1 (0 – 318)
Periventricular White Matter	26	7.7 (0–30.2)	71	53.9 (0 – 152)

### Radiation dose

**Fig. 2:**
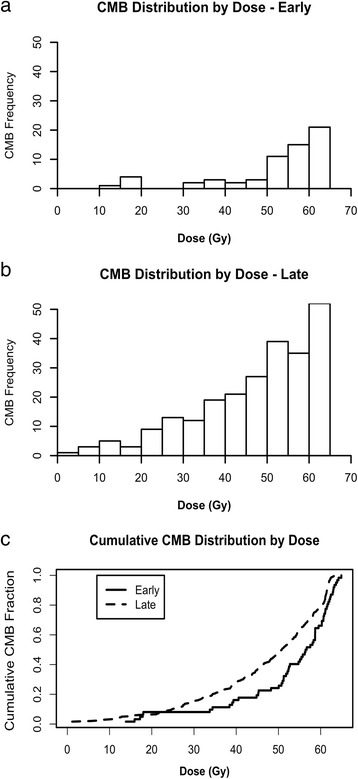
Distribution of microbleeds by dose. **a**, **b**) Histogram of microbleeds vs dose at the early (<3 years) and late (≥3 years) timepoints, respectively. **c**) Cumulative distribution function of microbleed formation by dose for early (solid) and late (dashed) timepoints. The distribution between the two timepoints was significantly different by a Kolmogorov-Smirnov test (*p* = 0.006)

The distribution of CMBs by dose is shown in Fig. 2; the vast majority of all CMBs (88%) were observed at doses over 30 Gy. However, the distribution of CMBs by dose was significantly different between early and late timepoints (*p* = 0.006, Fig. 2c), with a relative shift towards formation of CMBs at lower dose at the later timepoints. Binned into low (<30 Gy), medium (30–45 Gy) and high dose (>45 Gy) regions, the proportion of MBs in each dose region at the early timepoint was 3%, 12% and 85%, respectively; the corresponding proportions at the late timepoint was 15%, 22% and 64%, respectively; these proportions were significantly different (*p* < 0.001). At the early timepoint, only 2 of 9 patients exhibited microbleeds in the low dose region.

**Fig. 3:**
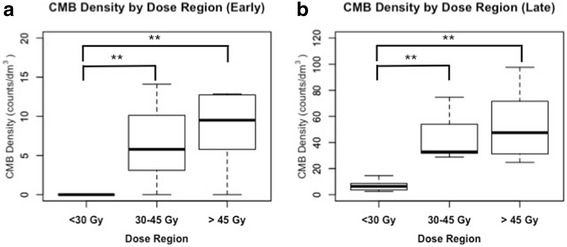
CMB density by dose. Boxplot of CMB densities for each subject in low, medium and high-dose regions at early (**a**) and late (**b**) timepoints. Significant differences between dose regions by Wilcoxon rank-sum tests are shown; *p < 0.05. **p < 0.01

To account for differences in brain size, the number of CMBs within each dose region was then normalized by the total volume of that region to generate a metric of microbleed density (MD). MD in each dose region for early and late timepoints are shown in Fig. 3. The density within each dose region was significantly higher at late timepoints (high: *p* < 0.001, medium: *p* = 0.001, low: *p* < 0.001). Comparing dose regions within each timepoint, the density of CMBs within the medium and high-dose regions were significantly higher than that in the low dose region (Early: medium vs low *p* = 0.006, high vs low *p* = 0.001; Late: medium vs low *p* < 0.001, high vs low *p* < 0.001). However, no significant difference in density between the medium and high-dose regions were observed (Early: medium vs high *p* = 0.33, Late: medium vs high *p* = 0.9).

The relationship between the number of CMBs and dosimetric parameters, including the volume of brain receiving at least 30 Gy (V30), 45 Gy (V45) and 60 Gy (V60) were examined. At early time points, the total number of CMBs seen in each patient was significantly correlated with the V30 (Spearman’s ρ = 0.68, *p* = 0.045) and V45 (Spearman’s ρ =0.78, *p* = 0.01), with a trend towards correlation with V60 (Spearman’s ρ = 0.63, *p* = 0.07). However, there were no significant correlations between V30, V45 or V60 and number of CMBs at later timepoints (Spearman’s ρ = 0.23, 0.31, −0.05 and *p* = 0.61, 0.50, 0.91, respectively).

### Anatomic location

The tumors were located primarily in frontal and temporal lobes (5 frontal lobe, 4 temporal lobe, 1 parietal lobe, 2 occipital lobe, 1 thalamic). The total number of CMBs observed in each cortical lobe is reported in Table 1. In order to account for variation in the distribution of radiation dose across the lobar volumes, the CMB count was normalized by the volume of each lobe receiving at least 30 Gy (V30) to calculate a microbleed density (MD_V30_). While qualitatively there appeared to be a lower microbleed density in frontal and occipital lobes, the distribution of MD_V30_ was not significantly different between lobes (*p* = 0.29 and *p* = 0.30 for early and late time points, respectively).

## Discussion

Adjuvant radiotherapy remains the standard of care for all patients with newly diagnosed WHO grade III and IV gliomas, and is increasingly used for patients with WHO grade II gliomas [1, 2]. Due to the large amount of normal brain tissue encompassed in radiation fields for gliomas, a major late complication of treatment is long-term cognitive decline. While progressive decline has been demonstrated in patients with glioma undergoing radiotherapy [7], particularly for those with WHO grade II gliomas with long overall survival, the patient and treatment factors that increase the risk of cognitive decline are not well defined. Cerebral microbleeds (CMBs) have been previously observed in patients undergoing radiotherapy [25–27, 29–31], and have been linked to cognitive decline [27, 28]. However, the precise relationship between radiation dose and CMB formation has not been characterized.

In this study, we performed the first quantitative assessment of the effect of radiation dose on microbleed formation, demonstrating a time-dependent relationship between microbleed formation and dose, with an increasing proportion of microbleeds forming in lower dose regions with increasing time from receipt of radiotherapy. This finding is consistent with prior qualitative observations that patients scanned at later timepoints are more likely to have microbleeds present in the cerebral hemisphere contralateral to their initially irradiated lesion [25]. The incidence of microbleeds increased significantly at all dose levels between 2 and 4 years after treatment, highlighting that microbleed formation represents a late sequelae of radiation that can take years to fully manifest, consistent with the clinically observed time course for the development of cognitive decline [37].

Collectively, these results are consistent with a dose-latency effect in CMB formation: CMBs appear to form with relatively short latency in high-dose regions, with longer latency seen in areas receiving lower radiation dose. While the precise radiobiologic mechanism for this dose-latency effect has not been elucidated, we hypothesize that different mechanisms for CMB formation may be involved in low and high-dose regions. Specifically, direct endothelial damage on a relatively short time scale may dominate in high-dose regions, while indirect effects on vasculature with a longer latency period, including vascular proliferation, perivascular fibrosis and chronic microglial activation, may dominate in low-dose regions [9]. Further study of this dose-latency effect is warranted.

Although there was a trend towards increased microbleed density over 45 Gy, the density within regions receiving moderate dose (30–45 Gy) was overall similar to that observed in high-dose regions (>45 Gy), compared to the reduced CMB density within low-dose regions (<30 Gy). These results suggest that the risk of CMB formation does not increase linearly with dose, particularly at later timepoints, arguing for a ceiling effect for CMB formation hypothesized in other studies [31], though a study with a larger cohort would be required to probe the dose–response relationship above 30 Gy in more detail. We also found that, at early timepoints, the total volume of brain receiving relatively low dose (V30 and V45) was a better dosimetric predictor of the risk of CMB formation than the high-dose region (V60). Thus V30 could be a useful metric clinically to assess risk of microvascular damage. Given that the 30 Gy volume was relatively large in patients undergoing radiotherapy for gliomas, these results highlight that areas at significant risk of CMB formation may be far from the site of the tumor and resultant high-dose region. Our findings are also consistent with the observed CMB formation and cognitive decline in patients undergoing whole brain radiotherapy, where a modest dose of 30 – 37.5 Gy (corresponding to 38–42 Gy in standard fractionation) is typically given [38, 39].

Although the density of CMBs within regions receiving under 30 Gy was relatively low initially, CMBs were still observed within these regions, particularly at later timepoints. These results are consistent with a prior study showing a high rate of CMB formation outside the region of high-dose radiation in pediatric patients [31]. Crucially, this study featured very long follow-up, with median follow-up of over 11 years, suggesting that the risk of CMB formation in low-dose regions may continue to increase for many years after radiotherapy, while a ceiling effect may prevent further formation of CMBs in high dose regions with longer follow-up. These findings together are of significant clinical importance, suggesting that pediatric patients undergoing low-dose radiotherapy, such as craniospinal radiation to a dose of 24 Gy for medulloblastomas, are at risk for late microvascular damage from treatment.

While we observed qualitatively fewer microbleeds in frontal and occipital lobe regions, we did not see a significant variation in microbleed density between lobar regions. However, this comparison may be confounded by the differing distribution of tumors within each lobe, and limited by our relatively small cohort. While the distribution of microbleeds in patients with Cerebral Amyloid Angiopathy demonstrated a specific lobar pattern of microbleed formation within temporal and occipital lobes [40], it is unclear whether there are underlying anatomic or functional differences between brain regions that confer variable susceptibility to microvascular damage by radiation.

In our study with a small patient cohort, we did not find any individual patient factors that were predictive of CMB formation. Specifically, no association between age or use of anti-angiogenic therapy was found. The lack of association with age has been seen in previous studies, but an association between anti-angiogenic therapy and decreased risk of CMB formation was previously observed [26]. However, our small cohort size limits any conclusions regarding individual patient risk factors for CMB formation.

Several limitations of our studies should be mentioned. First, our patient population was relatively heterogeneous, specifically with some patients undergoing experimental anti-angiogenic treatment, potentially limiting the generalization of our results. Second, the scan coverage of the entire brain was variable across the study, with images obtained early in the study lacking the full-brain coverage that was achieved with more robust parallel imaging routines implemented for more recent scans. For patients with microbleeds near the edge of the imaged volume, this limitation could potentially alter our estimation of both microbleed counts and the density of microbleeds within regions that were only partially evaluable for the presence of microbleeds. Third, since microbleeds were visually identified for each scan, there is the possibility of human error in lesion identification. It can be challenging to differentiate an en face vessel from a microbleed; however the contiguous slice coverage of the 3D scan and minimum-intensity projections limited this source of error. In addition, CMBs were iteratively counted until a consistent count was achieved. Finally, our study did not include longitudinal neurocognitive assessment for correlation with radiographic findings.

Further work is needed to better characterize CMBs and their clinical importance. First, while we demonstrate substantial patient variability in the rate of CMB formation, studies with larger cohorts are needed to identify specific patient factors that confer an increased risk of CMB formation. Second, the link between CMB formation and cognitive decline needs to be elucidated further. Specifically, the relationship between CMB formation in specific brain regions and cognitive decline is not well established, and it remains unclear whether CMB formation precedes the development of cognitive changes in a potentially clinically actionable manner.

## Conclusions

We have demonstrated dosimetric and temporal variability in the risk of formation of cerebral microbleeds after chemoradiotherapy for patients with malignant gliomas. Microbleeds formed predominantly within regions receiving at least 30 Gy of radiation without a clear dose–response relationship at higher doses, and increased in density with time from treatment. Our findings have important implications for the understanding of late microvascular sequelae of cranial irradiation, and may be clinically useful in assessing an individual patient’s risk of microbleed formation and resultant potential cognitive decline. Further work is needed to analyze the link between CMB formation and long-term cognitive changes in patients undergoing radiotherapy, and to determine individual patient factors that confer increased risk of CMB formation and long-term microvascular damage.

## Abbreviations

CMB: Cerebral microbleeds
CRT: Chemoradiotherapy
SPGR: Spoiled gradient echo
SWI: Susceptibility-weighted Imaging
WHO: World Health Organization
